# Travelling under pressure - hypoxia and shear stress in the metastatic journey

**DOI:** 10.1007/s10585-023-10224-8

**Published:** 2023-07-25

**Authors:** Ece Su Ildiz, Ana Gvozdenovic, Werner J Kovacs, Nicola Aceto

**Affiliations:** Department of Biology, Institute of Molecular Health Sciences, Swiss Federal Institute of Technology Zurich (ETH Zurich), Zurich, Switzerland

**Keywords:** Metastasis, Circulating tumor cell (CTC), Hypoxia, Hypoxia-inducible factors, Shear stress

## Abstract

Cancer cell invasion, intravasation and survival in the bloodstream are early steps of the metastatic process, pivotal to enabling the spread of cancer to distant tissues. Circulating tumor cells (CTCs) represent a highly selected subpopulation of cancer cells that tamed these critical steps, and a better understanding of their biology and driving molecular principles may facilitate the development of novel tools to prevent metastasis. Here, we describe key research advances in this field, aiming at describing early metastasis-related processes such as collective invasion, shedding, and survival of CTCs in the bloodstream, paying particular attention to microenvironmental factors like hypoxia and mechanical stress, considered as important influencers of the metastatic journey.

**Graphical abstract:**

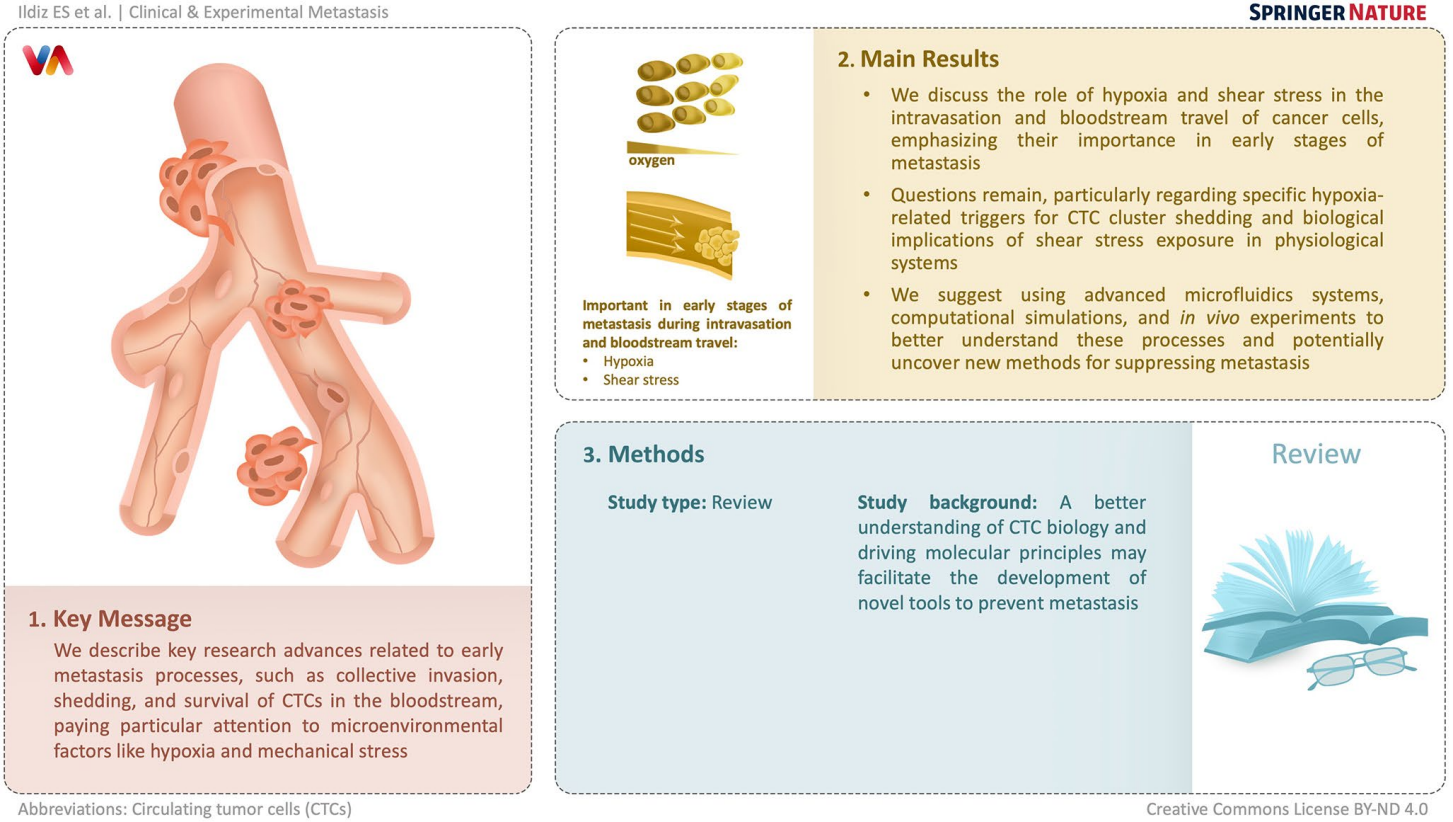

## Introduction

Metastasis encompasses the spread of cancer cells from the primary tumor to distant sites, *via* the bloodstream and/or the lymphatic system. Despite consistent advances in cancer treatment, metastatic disease remains largely uncurable and accounts for the vast majority of cancer-associated deaths. Improving our understanding of the molecular and cellular processes that drive metastasis, particularly in its early steps, is crucial for the development of effective treatments to prevent the spread of cancer.

Metastasis of solid tumors is a complex, multi-factorial process involving distinct sequential steps collectively termed as invasion-metastasis cascade. The major steps include cell migration, local invasion through adjacent tissues, intravasation into the bloodstream, arrest and extravasation, dissemination to adjacent or distant sites, survival and adaptation to the foreign microenvironment, establishment of micrometastases, and finally formation of clinically evident macrometastases [1]. Of note, these steps are continuously replicated, even in cells that have already metastasized, fostering metastasis-to-metastasis dissemination [2]. Invasion into the surrounding extracellular matrix (ECM) is one of the essential early steps in the metastatic cascade [3]. Histopathological examination of patient tumor specimens, intravital imaging in mouse cancer models and in vitro experimental systems revealed that tumor cells can display diverse invasive behaviors [4, 5]. Broadly speaking, three major categories can be distinguished: cells can employ either amoeboid or mesenchymal single cell invasion, multicellular streaming (elongated strands of loosely-attached tumor cells moving through a common path) or collective invasion, characterized by sustained cell-cell adhesion [5]. Modes and dynamics of cancer cell invasion are not merely regulated by intrinsic cancer cell factors, but also depend on multiple elements of the tumor microenvironment (TME) [6]. In addition to cancer cells, the TME comprises a broad range of non-malignant cell types, including immune cells, cancer-associated fibroblasts (CAFs), endothelial cells, pericytes, and various other tissue-specific cell types that are all surrounded by vascularized and modified ECM [7].

It is increasingly acknowledged that collective cell invasion, including the formation of cell clusters, is a key mechanism in the progression of solid tumors. Two types of cells can be identified among collectively invading units: leader and follower cells. Leader cells, either tumor-derived or stroma-derived, are in charge of creating low resistance tracks to be exploited for migration, both by using biochemical and biomechanical mechanisms, such as matrix deposition, proteolysis and cytoskeletal remodeling. Leader cells can be classified into four main categories: mesenchymal-like (or hybrid epithelial/mesenchymal) tumor cells, basal epithelial tumor cells, CAFs and tumor-associated macrophages (TAMs) [4]. In contrast to highly invasive leader cells, follower cells are most frequently described as a phenotypically distinct subpopulation of cancer cells, characterized by low invasive potential and migrating along the created invasive paths. The ways by which leader cells collectively coordinate both leader and follower cells, as well as their specific contribution to metastasis remains unclear. Cancer cells that leave the primary tumor and enter the circulation are referred to as circulating tumor cells (CTCs), and act as first-line pioneers of the metastatic cascade [8]. While the primary tumor is thought to shed large numbers of tumor cells, only an extremely small fraction of CTCs will effectively give rise to secondary tumors [3, 9, 10]. CTCs circulate in the blood as single cells or as aggregated cell clusters formed by two or more tumor cells (homotypic CTC clusters) or formed by tumor cells and non-neoplastic cells (heterotypic clusters) [11–14]. Multicellular CTC clusters have a higher metastatic potential compared to individual CTCs, and their occurrence in patients with various cancer types is linked to a poor prognosis [14, 15].

In this review, we discuss research advances in the field of early metastasis events, with a particular focus on the mechanistic determinants of collective invasion, shedding and survival of CTCs in the bloodstream. Particularly, we will highlight hypoxia and mechanical stress as key regulators of CTC biology and metastatic proclivity.

### Hypoxia in the tumor microenvironment

**Fig. 1:**
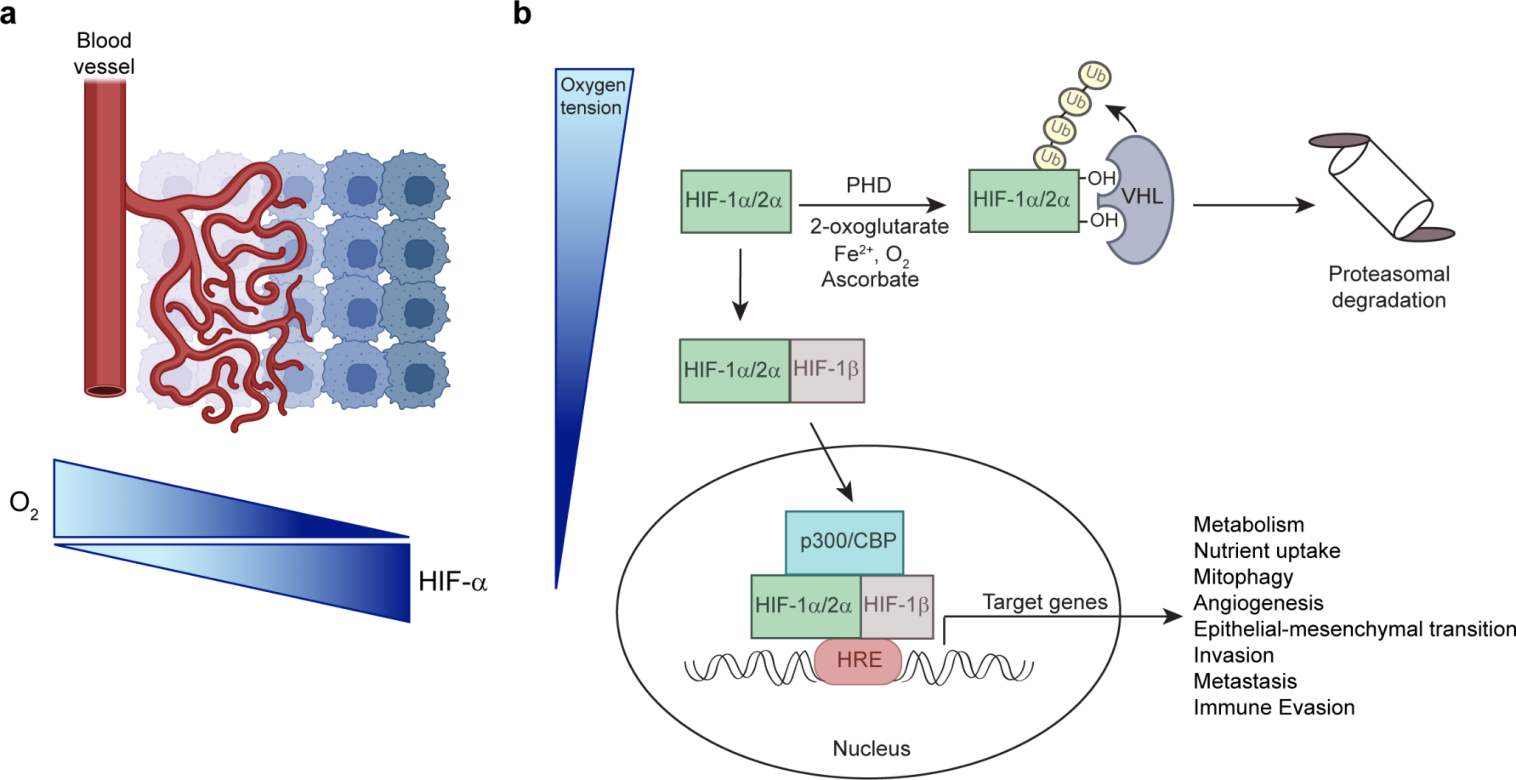
(**a**) In solid tumors, HIF-α is stabilized under conditions of low O_2_ due to reduced vascularization and the establishment of a hypoxic microenvironment. (**b**) Post-transcriptional regulation of HIF-α subunits. Under normoxic conditions, PHD enzymes utilize oxygen and 2-oxoglutarate as substrates to hydroxylate two proline residues in the HIF-α subunit. These hydroxylation events are required for VHL to bind, ubiquitinate, and target HIF-α for proteasomal degradation. Under hypoxia, hydroxylation is inhibited and HIF-α stabilized. HIF-α heterodimerizes with HIF-1β, interacts with the transcriptional coactivators p300 and CBP, and binds to HRE elements within regulatory regions of target genes. Illustrations were created with BioRender.

Hypoxia, or low oxygen (O_2_) tension, is a key factor of the TME that influences the behavior of both tumor and stromal cells. Studies measuring the partial pressure of O_2_ (pO_2_) in solid tumors have shown a median pO_2_ value of ~ 10 mm Hg, as compared with for example 65 and 42 mm Hg in normal human breast and cervix tissue, respectively [16, 17]. pO_2_ values < 10 mm Hg have been associated with a worse prognosis [17, 18]. Two types of hypoxia are observed in tumors, namely chronic and cycling (intermittent) hypoxia (Fig. 1a). Chronic hypoxia is defined as O_2_ deficiency over a continuous period of time (at least several hours) and affects cells that are rather distant from blood vessels. In order to grow beyond a certain size, tumors require nourishment in the form of nutrients and O_2_, as well as an ability to remove metabolic wastes and carbon dioxide. Therefore, during tumor progression, an angiogenic switch is activated that boosts vascular supply by causing the normally quiescent vasculature to continually sprout new blood vessels [19–21]. However, the tumor vasculature is not fully functional; due to an improper balance and/or excessive production of angiogenic factors, it is chaotically organized, leaky, and blood often follows different paths through the same vessel [22–25]. The abnormal tumor vasculature leads to irregular O_2_ perfusion of the tumor tissue and therefore tumors experience temporal and spatial fluctuations in oxygenation [22, 26, 27]. This type of hypoxia is named cycling hypoxia and affects cells immediately adjacent to inefficiently perfused blood vessels, and when the blood flow is restored, the hypoxia period is followed by a reoxygenation period. Reoxygenation can cause “reoxygenation injury” to the cells, involving free radical formation, reactive oxygen species (ROS) generation, and tissue damage [26, 28].

Hypoxia-inducible factors (HIFs) are transcription factors that are central to the molecular mechanisms underlying O_2_ homeostasis and function as master regulators of the adaptive response to hypoxia. HIF-α signaling regulates multiple specific biological processes that are involved in many stages of the metastatic cascade, and the focus of the following sections is on the hypoxia-related steps leading up to the intravasation of cancer cells.

### Regulation of hypoxia-inducible factors

HIFs are frequently overexpressed in human cancers because of either intratumoral hypoxia or genetic alterations that lead to HIF-α stabilization (e.g., loss of the von Hippel-Lindau (VHL) tumor suppressor) [29]. HIFs are heterodimers containing an O_2_-sensitive HIF-α subunit and an O_2_-independent, constitutively expressed aryl hydrocarbon receptor nuclear translocator (ARNT)/HIF-1β subunit. Three HIF-α subunits have been identified, namely HIF-1α, endothelial PAS domain protein 1 (EPAS1)/HIF-2α, and HIF-3α [29]. HIF-1α is ubiquitously expressed, while HIF-2α is expressed in endothelial cells and in distinct cell populations of kidneys, brain, lungs, liver, gastrointestinal tract, pancreas, and heart [30]. The *HIF3A* gene gives rise to multiple HIF-3α isoforms by utilizing different promoters, different transcription initiation sites, and alternative splicing [31]. The multiple HIF-3α isoforms have different and even opposite functions, and studies suggest that they negatively regulate the activity of HIF-1α and HIF-2α [31, 32]. Under normoxia, HIF-α subunits are hydroxylated by prolyl hydroxylase domain (PHD)-containing enzymes on two proline residues within the O_2_-dependent degradation domain (Fig. 1b) [32]. The hydroxylated HIF-α subunits are recognized and targeted for proteasomal degradation by the VHL E3 ubiquitin ligase complex [33]. Hypoxia or oncogenic alterations such as loss of function of VHL, phosphatase and tensin homolog (PTEN), tumor protein 53 (TP53) as well as activation of the phosphoinositide 3-kinase (PI3K)-AKT pathway stabilize HIF-α subunits [34–36]. The stabilization of HIF-α subunits under non-hypoxic conditions is a phenomenon termed pseudohypoxia [29]. The HIF-α subunits dimerize with HIF-1β and interact with the transcriptional coactivator p300/cAMP response element-binding protein (CREB)-binding protein (CBP) complex. This transcriptional complex binds to hypoxia-response elements (HREs) in promoters of HIF-α target genes involved in metabolism, nutrient uptake, apoptosis resistance, sustained growth factor signaling, replicative immortality, invasion, metastasis, angiogenesis, and erythropoiesis [29, 37, 38]. HIF-1α and HIF-2α have both overlapping and distinct target genes [32].

In general, HIF signaling reinforces most hallmarks of cancer [20, 21] and confers cancer cells with more aggressive characteristics in hypoxic niches. Increased expression of HIF-1α and/or HIF-2α has been associated with increased tumor aggressiveness and poor prognosis in a broad range of tumor types [39]. Increased levels of HIF-1α, which correlate with poor prognostic outcomes and increased metastasis in patients, have been identified in many solid tumors such as breast, cervical, ovarian, pancreatic, gastric, colorectal, esophageal, lung, liver and prostate cancer [40–45]. In breast cancer, survival is significantly decreased in patients with the highest HIF-1α levels, regardless of the lymph node status [46, 47]. HIF-2α expression in primary tumors is associated with distant metastasis and poor outcome in patients with small cell lung cancer, non-small cell lung cancer, clear cell renal cell carcinoma (ccRCCs), neuroblastoma, and breast cancer [48–52]. Intratumoral hypoxia is associated with the invasion and metastasis of HIF-1α-active pancreatic cancer cells [53, 54], and eradication of the HIF-1α-active cells compromises malignant progression. In addition, it has been shown that highly metastatic pancreatic ductal adenocarcinoma (PDAC) subpopulations are enriched for hypoxia-induced genes, and hypoxia-mediated induction of the transcription factor B lymphocyte-induced maturation protein-1 (BLIMP1) contributes to the regulation of a subset of hypoxia-associated gene expression programs [55]. ccRCC is the most common subtype (~ 75%) of renal cancer and complete loss of VHL function occurs in ~ 90% of ccRCCs, leading to constitutive stabilization of the HIF-α subunits and activation of their signaling [51, 52]. HIF-2α is considered to be a driver oncoprotein for ccRCC. Almost a third of all patients with ccRCC show metastatic dissemination at presentation (at which time it has 95% mortality), and ~ 60% have metastases within the initial 2–3 years after diagnosis. Furthermore, it has been shown in experimental models that overexpression of HIFs in several tumor cell types promotes metastasis, whereas inactivation of HIFs decreases the metastatic potential of tumor cells [56].

Several studies have shown that HIF-1α upregulation is more strongly induced by repeated exposures to hypoxia–reoxygenation than by chronic hypoxia [57–61]. The primary consequence of cycling hypoxia is upregulation of HIF-1α activity to a level that supersedes that of chronic hypoxia. The mechanism of enhanced HIF-1α activity is multifactorial, but one factor is the hypoxia-reoxygenation injury-induced production of ROS, which stabilize HIF-1α even in the presence of enhanced oxygenation. In contrast to HIF-1α, the effect of cyclic hypoxia on the stabilization of HIF-2α has been understudied. One study even showed that cyclic hypoxia leads to the degradation of HIF-2α *via* a calpain-dependent signaling pathway, which in turn results in oxidative stress [62].

### HIF-mediated metabolic reprogramming

In the last few years, several studies have addressed how tumor acidosis participates in cancer progression. In hypoxia, cells shift from O_2_-dependent mitochondrial adenosine triphosphate (ATP) production to O_2_-independent production, *via* glycolysis. In cancer cells, the rate of glucose uptake is dramatically increased and lactate is produced, even in the presence of oxygen and fully functioning mitochondria. This process is known as the Warburg Effect or aerobic glycolysis and required for tumor growth [63]. The rate of glucose metabolism through aerobic glycolysis is higher compared to mitochondrial respiration, and the production of lactate is 10–100 times faster than the complete oxidation of glucose in the mitochondria [63]. In addition, the Warburg effect supports the biosynthetic requirements of proliferating cells by diverting glucose-derived carbon into the multiple branching pathways of glycolysis [64].

**Fig. 2:**
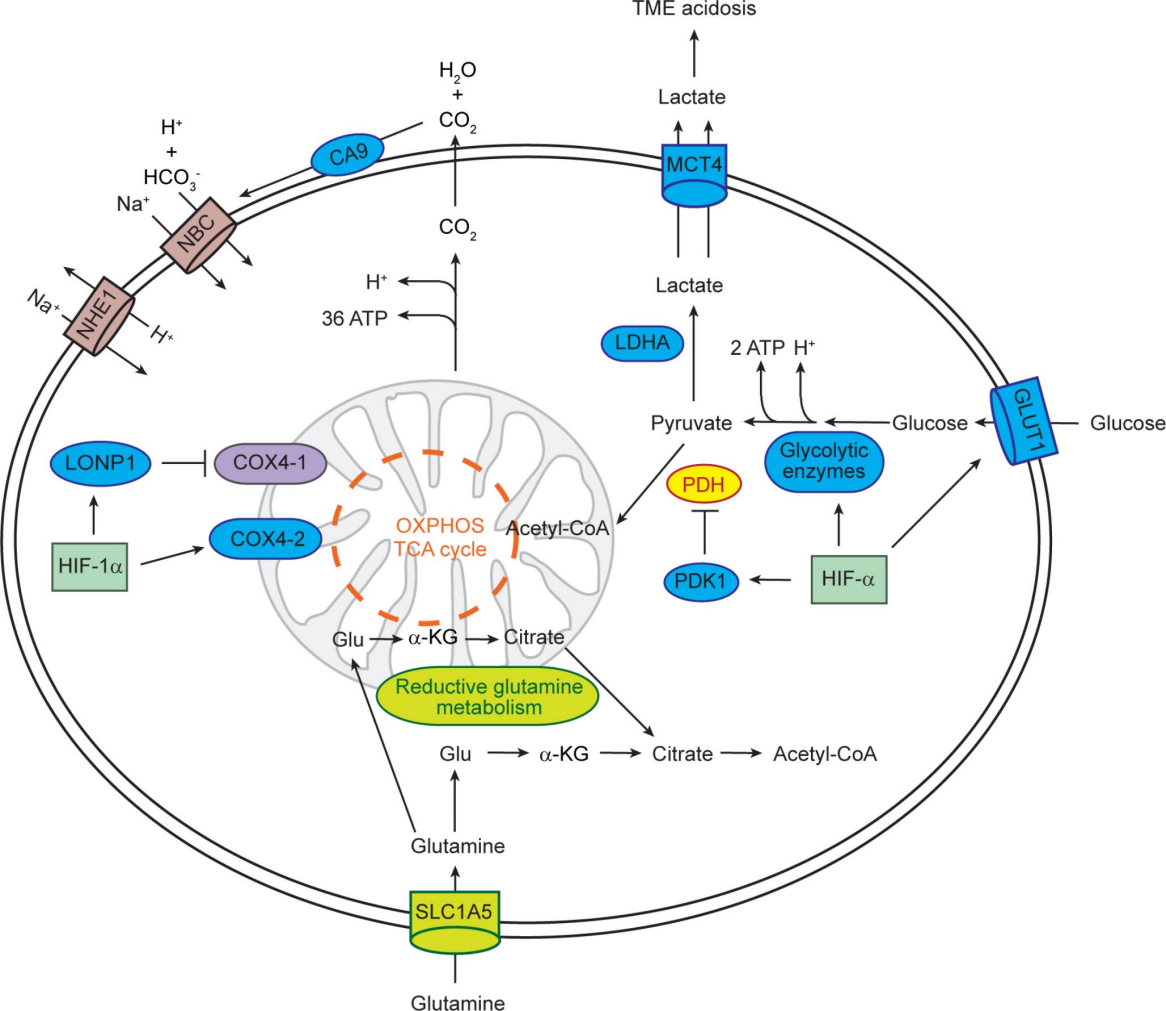
Regulation of glucose and glutamine metabolism and pH by HIF-α. Blue circles indicate proteins encoded by HIF-α target genes. To compensate for the reduced flux of glucose to citrate, reductive glutamine metabolism generates cytosolic citrate for *de novo* lipid synthesis. α-KG, α-ketoglutarate; Glu, glutamate; OXPHOS, oxidative phosphorylation; NBC, Na^+^/HCO_3_^−^ co-transporter; TCA, tricarboxylic acid

HIF signaling enhances glycolysis by inducing genes that encode glucose transporters (e.g., *GLUT1/SLC2A1*, *GLUT3/SLC2A3*) and glycolytic enzymes (Fig. 2) [65]. Lactate dehydrogenase A (*LDHA*), which converts pyruvate to lactate and ensures NAD^+^ regeneration for glycolysis, and monocarboxylate transporter 4 (*MCT4*), which transports lactate and H^+^ out of the cell, are also upregulated by HIF signaling (Fig. 2). Remarkably, lactate produced by hypoxic cancer cells can be taken up by non-hypoxic cancer cells or stromal cells via MCT1 to regenerate pyruvate, used for oxidative phosphorylation [66, 67]. Consequently, glucose freely diffuses through the oxygenated tumor cell sheath to fuel glycolysis of hypoxic tumor cells.

Cancer cells have evolved several mechanisms to maintain their intracellular pH [68]. H^+^ can also be exported by specific H^+^ transporters, including Na^+^/H^+^ exchanger 1 (NHE1/SLC9A1) [68]. Hypoxic cancer cells counteract local acidosis by HIF-dependent induction of the membrane-bound ectoenzyme carbonic anhydrase 9 (*CA9*), which converts H_2_O and metabolically generated CO_2_ to H^+^ and HCO_3_^−^ (Fig. 2) [69, 70]. Na^+^/HCO_3_^−^ co-transporters facilitate HCO_3_^−^ flux through the cell membrane to maintain the alkaline intracellular pH. In summary, the activity of MCT4, NHE1, and CA9 leads to intracellular alkalization of cancer cells.

HIF signaling suppresses both the tricarboxylic acid cycle (TCA) and oxidative phosphorylation within mitochondria (Fig. 2). HIF-α induces the expression of pyruvate dehydrogenase kinase 1 (*PDK1*), which phosphorylates and inhibits the mitochondrial pyruvate dehydrogenase (PDH), thereby inhibiting the conversion of pyruvate to acetyl-CoA for entry into the TCA cycle [71, 72]. A HIF-1α-mediated isoform switch from cytochrome c oxidase subunit 4 isoform 1 (COX4-1) to COX4-2 optimizes the efficiency of mitochondrial respiration under hypoxia [73]. The mitochondrial Lon peptidase 1 (*LONP1)* is induced by HIF-1α and degrades COX4-1. Attenuation of mitochondrial metabolism and O_2_ consumption in response to hypoxia is also achieved by HIF-α-mediated suppression of mitochondrial biogenesis and enhancement of selective mitochondrial autophagy (mitophagy) [29].

A common feature of cultured cancer cells is glutamine addiction [74]. Under conditions where HIF-α is stabilized, cells shift from oxidative to reductive glutamine metabolism and reverse TCA cycle flux to compensate for the reduced flux of glucose to citrate (Fig. 2) [29]. Glutamine is metabolized through glutaminolysis to α-ketoglutarate, which is converted to citrate by isocitrate dehydrogenase 1 or 2 and aconitase [75]. Thus, reductive glutamine metabolism is the major source of acetyl-CoA for *de novo* lipid synthesis when HIF-α is stabilized.

The increased consumption of glucose and glutamine by cancer cells causes metabolic competition for nutrients in the TME between cancer cells, stromal and immune cells, ultimately promoting cancer progression [76]. It can negatively impact the functions of immune cells in the TME such as T cells, TAMs, and myeloid-derived suppressor cells (MDSCs) [77]. TAMs and T cells depend on glucose availability and glucose metabolism. Naïve T cells in a resting state require low amounts of glucose, amino acids, and fatty acids to meet basic energy requirements, but active T cells increase glucose and glutamine catabolism for nucleotide and lipid synthesis as well as ATP production [78]. Tumor cells, macrophages, and tumor-infiltrating lymphocytes (TILs) compete for glucose in the TME, and the high rates of glycolysis in tumor cells limit the availability of glucose to TAMs and TILs, which require sufficient glucose for their effector functions [76, 79]. High lactate concentrations in the TME disturb the metabolism and function of human cytotoxic T lymphocytes and suppress their proliferation and cytokine production [80]. Tumor-derived lactate also suppresses survival and function of TILs and natural killer (NK) cells, leading to immune evasion [81]. The intracellular acidification blocks the interferon-γ production in T and NK cells by preventing the upregulation of the transcription factor nuclear factor of activated T cells (NFAT). High rates of cancer cell glycolysis suppress anti-tumor T cell effector functions by depriving T cells of glucose and the downstream metabolite phosphoenolpyruvate, which regulates Ca^2+^-NFAT signaling in T cells [79].

TAMs promote tumor cell invasion, intravasation, and induce angiogenesis in primary tumors [82]. The extracellular acidification increases the proteolytic activity of TAMs to enhance cell motility [21, 83]. Cancer cell-derived lactate leads to HIF-1α-dependent polarization of TAMs towards a tumor-permissive M2 phenotype and induces vascular endothelial growth factor A (*VEGFA)* expression in TAMs [84]. In breast cancer, a subpopulation of perivascular TAMs has been identified that has high levels of the TEK receptor tyrosine kinase and expresses VEGFA, which increases vasculature leakiness and causes tumor cell intravasation [85]. In addition, HIF-1α-mediated proangiogenic signaling in the TME also occurs in breast CAFs [86].

### HIF signaling and invasion

#### Acid-mediated invasion

The increased glucose metabolism in cancer cells decreases the pH in the TME due to lactate secretion, and the acidity results in increased progression and metastasis [87–89]. A lactate gradient is formed in the TME with the highest concentration in the most hypoxic regions. In a study using breast and colon cancer cells, invasion and peritumoral pH were monitored using intravital microscopy [87]. The peritumoral pH is acidic and heterogeneous and the regions of highest tumor invasion corresponded to areas of lowest pH, whereas no invasion is evident in regions with normal or near-normal extracellular pH [87]. An examination of core and invasive edges of invasive ductal breast carcinoma showed that tumor edges are characterized by increased staining of HIF-1α, CA9, and GLUT1 [90]. Interestingly, the vascular marker CD34 is co-expressed with these hypoxia markers, suggesting that they might be expressed in well-oxygenated regions. In addition, with the acidosis markers - the pH-(low)-insertion peptides (pHLIP), plasma membrane-localized lysosome-associated membrane protein 2 (Lamp2), and CA9 it has been shown that acidic regions are not restricted to hypoxic areas, yet overlap with highly proliferative, invasive regions at the tumor-stroma interface [91]. These and other studies led to the acid-mediated invasion model [89]. Accordingly, several studies have shown that neutralization of tumor acidosis can inhibit metastasis [89].

The increased motility of cancer cells is due in part to changes in the dynamics of the cytoskeleton. Cancer cells have a slightly higher intracellular pH (pH_i_ >7.4) than normal cells (pH_i_ ~7.2), which leads to *de novo* assembly of actin filaments through a pH-dependent increase in the activities of several actin-binding proteins [68, 90]. Hypoxia also promotes remodeling of the actin cytoskeleton and cancer cell motility through the activation of the RhoA/Rho-associated protein kinase (ROCK) signaling pathway by HIF-1α, which leads to increased invasion and migration of hepatocellular carcinoma (HCC) cells in vitro and in vivo [92].

#### Hypoxia-induced ECM remodeling

Cell migration and invasion require the remodeling of ECM structures. For invasion to occur, cancer cells must degrade the surrounding basement membrane (BM). HIF signaling promotes ECM remodeling through the upregulation and secretion of proteolytic enzymes. Matrix metalloproteinases (MMPs) are the main enzymes involved in ECM degradation, and increased levels of many MMPs in both primary tumors and/or metastases are positively associated with tumor progression [93]. HIF signaling induces the expression of *MMP1*, *MMP2*, *MMP3*, *MMP9*, *MMP13*, *MMP14*, and *MMP15* [94–97]. It has been shown that MMP2 and MMP14 are expressed selectively in leader cells during collective invasion [98], and the knockdown of *MMP14* inhibited fibrosarcoma and breast cancer cell collective invasion [99]. The HIF-dependent upregulation of the urokinase plasminogen activator surface receptor (*PLAUR*) also increases the proteolytic activity of cancer cells, thereby altering the interaction between integrins and the ECM, promoting cell invasion [100–102].

The tissue inhibitors of metalloproteinases (TIMPs) family consists of four members and controls the enzymatic activity of MMPs [95, 103]. The HIF-dependent downregulation of TIMPs has been shown to increase the invasion ability of cancer cells and enhance metastasis [104–106].

In addition to the perforation of the BM by proteolytic enzymes, CAFs assist cancer cells in breaching the BM to promote invasion. CAFs can also facilitate BM breakthrough in a MMP-independent manner, by applying physical forces to the BM and by widening of pre-existing gaps initially created by MMPs or other proteases such as PLAUR [107].

Solid tumors are often characterized by excessive deposition of ECM proteins (referred to as fibrosis), and therefore are often stiffer than the surrounding normal tissue [108–111]. Changes in the posttranslational modification, deposition, and degradation of the matrix result in changes in the composition, density, and mechanical properties of the ECM [112, 113], which in turn affect ECM stiffness, cell migration, tumor growth, invasion, and metastasis [114–116]. Hypoxia has been shown to promote fibrosis in breast cancer [117, 118] and PDAC [119–122]. Chronic hypoxia is an important determinant of fibrosis and carcinogenesis in the liver [123, 124]. In addition, breast cancer and PDAC aggression associate with a stiffer ECM [113].

The HIF pathway controls the expression of genes encoding collagens and collagen-modifying enzymes [125, 126]. Posttranslational modifications of collagen include the hydroxylation of proline and lysine residues that are catalyzed by prolyl-4-hydroxylase α-subunit isoform 1 (P4HA1) and 2 (P4HA2) and procollagen-lysine 2-oxyglutarate 5-dioxygenase 1 (PLOD1) and 2 (PLOD2) [113, 127, 128]. These enzymes catalyze modifications that are necessary for the production of stiff and aligned collagen fibrils. HIF-1α induces the expression of *P4HA1*, *P4HA2*, *PLOD1*, and *PLOD2* in different cancer and non-cancer cell lines [109, 129–136], and enhanced activities of these enzymes are associated with increased collagen deposition in cancer tissue. In sarcoma, increased expression of *PLOD2* is associated with a more metastatic phenotype, and loss of HIF-1α and HIF-1α-dependent *PLOD2* expression disrupts cell migration and pulmonary metastasis [133]. Hypoxia-induced expression of *PLOD2* in sarcomas enhances collagen fiber size and tumor density and thus promotes lung metastasis [137]. The knockdown of *PLOD2* also impairs the invasion of breast cancer cells into the adjacent normal tissue of the mammary fat pad [136]. Knockdown of *P4HA1*, *P4HA2*, and *PLOD2* inhibits the spontaneous metastasis of breast cancer cells to the lungs and lymph nodes of mice [135, 136, 138].

ECM stiffening is induced by increased collagen crosslinking, which is mediated by lysyl oxidase (LOX) and LOX-like (LOXL) enzymes [139]. The expression and secretion of LOX, LOXL2, and LOXL4 is induced under hypoxia by HIF-1α [125, 140–145]. The ECM remodeling by LOX and LOXL enzymes leads to collagen fiber realignment, bundling, and stiffening with dense collagen bundles that are frequently positioned perpendicular to the tumor, thereby allowing cancer cells to migrate *via* the fibers and invading the host tissue [146–148]. Hypoxic secretion of LOX family members by cancer cells also regulates the formation of premetastatic niches in distant organs, priming these sites for colonization by metastatic cancer cells [56, 109, 111, 139]. Hence, HIF-driven ECM remodeling also has long-distance effects.

An important mediator of cell invasion is the hepatocyte growth factor (HGF) that acts through the MET tyrosine kinase receptor (MET) [149, 150]. HGF is a pleiotropic cytokine and hypoxia sensitizes cells to HGF stimulation by inducing the expression of MET [151, 152]. For example, in non-small-cell lung cancer, it has been shown that prolonged treatment with the MET inhibitor JNJ-605 and the epidermal growth factor receptor inhibitor erlotinib resulted in aggravation of Warburg metabolism in cancer cells with increased lactate release [153].

The gene encoding the C-X-C motif chemokine receptor 4 (*CXCR4)* is a direct HIF target [154]. CXCR4 is highly expressed in many types of cancer cells, and elevated CXCR4 levels correlate with distant metastases, poor prognosis, and unfavorable outcomes in most solid tumors [155, 156]. HIF signaling also regulates *CXCR4* expression in endothelial cells [157]. The upregulation of CXCR4 also aids in the transendothelial migration of cancer cells. A study suggested that hypoxia could drive intravasation via signaling through CXCR4 and its ligand stromal cell derived factor 1 (SDF-1, also known as C-X-C motif chemokine ligand 12 (CXCL12)), which results in adhesion of cancer cells to endothelial cells and trans-endothelial migration of breast cancer cells in in vitro assays [157]. Accordingly, neutralizing the interactions of CXCL12/CXCR4 impaired metastasis of breast cancer cells to regional lymph nodes and lung [158].

Extensive in vitro studies have shown that the migration of cancer cells is associated with an epithelial-to-mesenchymal transition (EMT), whereby cells lose expression of epithelial markers such as E-cadherin and increase expression of mesenchymal markers (e.g., N-cadherin, fibronectin, and vimentin) [159]. This leads to changes in cell-cell and cell-matrix adhesions. Hypoxia and HIF signaling have been linked to EMT-like phenomena by the induction of the transcription factors such as twist family BHLH transcription factor (*TWIST)* [160], snail family transcriptional repressor 1 (*SNAI1)* [161–163], zinc finger E-box binding homeobox 1 (*ZEB1)* [164], and snail family transcriptional repressor 2 (*SNAI2*) [165], and the modulation of cell signaling pathways such as neurogenic locus notch homolog protein (NOTCH), WNT, and AXL [166–172]. However, the consensus that EMT is required for cancer metastasis has been challenged by studies showing that CTCs are not the homogeneous mesenchymal phenotype described in the EMT-metastasis hypothesis [173]. A study using a mouse lineage-tracing model suggested that cells do not activate EMT in order to metastasize [174]. Along these lines, absence of TWIST1 or SNAI1 did not alter invasion and metastasis in genetically engineered PDAC mouse models [175]. Recent studies suggest that instead of being a binary process, EMT rather occurs through distinct intermediate states, a process referred to as hybrid EMT [176, 177]. Multiple subpopulations of cancer cells have been identified that are associated with different states of EMT, ranging from epithelial to mesenchymal-like states [178]. EMT or mesenchymal-to-epithelial transition (MET) are therefore not “all-or-none” processes, and cells undergoing hybrid EMT may display cellular plasticity associated with distinct invasive and metastatic potential [176, 178]. It has been suggested that the acquisition of a hybrid EMT state is involved in the determination of leader and follower cells [176].

#### CTCs and hypoxia

In the laboratory setting, a standard O_2_ level of 20% (160 mm Hg) used for cell cultures is referred to as normoxia. However, the physiological O_2_ levels (physoxia) found in normal tissues are much lower and average about 5% O_2_ (38 mm Hg) and range from ~ 3–7% [179]. It has been suggested that physiological hypoxia, the O_2_ level at which tissues respond to maintain their preferred O_2_ level, ranges from 2 to 6%, whereas O_2_ levels < 2% are referred to as pathological hypoxia [179]. O_2_ levels of 5% mimic more closely in vivo conditions and are therefore commonly utilized for expansion of patient-derived specimens in the laboratory. Along with dedicated media components, physiological hypoxia has been used to expand and maintain patient-derived CTCs ex vivo [180, 181]. While most of the laboratories employ hypoxic conditions, which have been proven critical for certain cancer types [180, 183], some CTC lines have been occasionally cultured under normoxic conditions as well [184].

Clinically, hypoxia and the expression of HIF-α are associated with increased distant metastasis and poor survival in a variety of tumor types [182]. Interestingly, in mouse models of breast cancer, a bimodal distribution of hypoxia has been observed, either restricted within a central core or more scattered throughout the tumor, often in areas with low blood vessel density [180]. Blood vessels are distributed in both normoxic and hypoxic regions of tumors, with a higher presence in normoxic tumor regions. The presence of functional blood vessels in hypoxic tumor areas indicates possible access routes for metastatic cells to the circulatory system. Furthermore, scattered hypoxic areas are due to cycling hypoxia, and cancer cells in these regions might be more prone to intravasation since they are located closer to blood vessels. It has been shown that intra-tumor hypoxia in breast cancer leads to upregulation of cell-cell junction components and intravasation of hypoxic CTC clusters with high metastatic ability, while single CTCs rather derive from normoxic tumor regions [180]. Anti-angiogenic therapies targeting the VEGF pathway have been developed to reduce intratumoral vasculature and consequently starve the tumor from its nutrients. However, in breast cancer patients, VEGF inhibitors lacked efficacy in the long run, even promoting tumor invasiveness and metastasis in some cases [23]. This led to withdrawal of the VEGF-targeting agent Avastin for the treatment of breast cancer patients in 2011 [183]. Accordingly, targeting VEGF in breast cancer mouse models leads to primary tumor shrinkage, but it increases intra-tumor hypoxia and results in a higher CTC cluster shedding rate and metastasis formation [180]. In contrast, pro-angiogenic treatment increases primary tumor size, but it dramatically suppresses the formation of CTC clusters and metastasis [180].

Using a fate-map system for hypoxic cells in vivo, cells exposed to physiological levels of hypoxia in the primary breast tumor were shown to have a four times greater probability of becoming viable CTCs, compared to cells from normoxic regions of the primary tumor [184]. Cells exposed to hypoxia in the primary tumor have the ability to migrate toward a more oxygenated invasive front of tumor regions and intermingle with blood vessels. In addition, post-hypoxic cells have a 6-7-fold greater probability of forming lung metastasis, suggesting that they have an enhanced metastatic potential [184]. Another study showed that oxidative stress prevents the survival of CTCs in the bloodstream and is therefore a limiting step in metastasis [185]. Cells that experience hypoxia in the primary tumor are more resistant to oxidative stress and have lower levels of mitochondrial ROS in both the primary tumor and in the blood [184]. It has been suggested that the enhanced metastatic potential of post-hypoxic cells is in part due to the ROS-resistant phenotype that allows them to survive high levels of ROS in the circulation.

Taken together, these observations highlight a prominent role of hypoxia in the early steps of the metastatic cascade, conferring both advantageous metabolic features as well as invasive traits that result in the intravasation of aggressive precursors of metastasis.

### Fluid shear stress

**Fig. 3:**
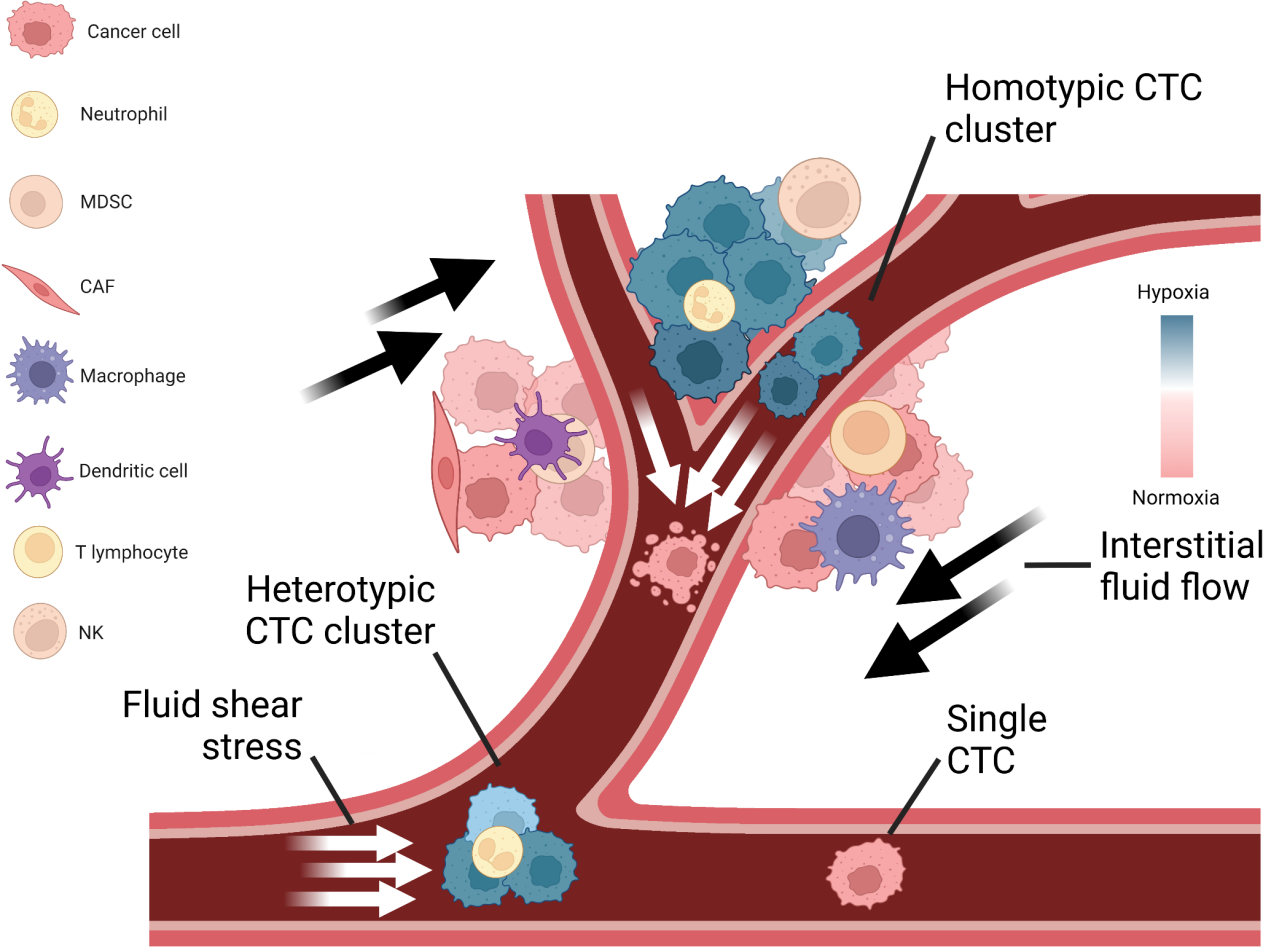
Hypoxia and shear stress regulate dissemination of circulating tumor cells (CTCs). Cancer cells are in interaction with several types of immune cells and experience various biophysical forces both in the primary tumor microenvironment and in the circulation. Primary tumors have normoxic and hypoxic regions with constant interstitial flow, inducing biochemical and biophysical signaling changes in cancer cells. Cancer cells can intravasate as single cells, as a group of cells (homotypic clusters) or together with non-neoplastic cells (heterotypic clusters). When CTCs enter the bloodstream, they are subjected to different levels of fluid shear stress that can lead to cell rupture and apoptosis, and initiate rapid signaling changes. Due to mechanical shielding, CTC clusters are more resistant to shear stress than single cells. In contrast, single cells are more prone to cell membrane blebbing caused by mechanical forces. MDSC, myeloid-derived suppressor cell; CAF, cancer-associated fibroblast; NK, natural killer cell. Illustrations were created with BioRender.

In addition to biochemical signals, biomechanical forces also affect tumor progression (Fig. 3). The effects of mechanical forces on living tissues are in the focus of mechanobiology, a multidisciplinary field at the interface between biology, physics and engineering that investigates how cells sense and respond to physical cues, and how these mechanisms impact their behavior and organization [186]. The field of mechanobiology is growing rapidly thanks to imaging techniques such as atomic force microscopy, technological innovations in force-application methods used to manipulate cells mechanically, microfluidics and advanced computational simulation [186–189]. Within the field, an extensively studied aspect is mechanotransduction, a process whereby cells convert forces arising from mechanical stimuli into biochemical signals. It involves sensing various physical stimuli through specialized ion channels, integrins and cytoskeletal structures, subsequently triggering alterations in gene expression, protein synthesis and cellular behavior [190]. Several types of solid stresses such as compression, tension, shear and ECM stiffness are applied to tumor cells from their environment [191]. It is widely recognized that these mechanical forces play a significant role in various steps of the metastatic cascade, starting from the growth of the primary tumor, intravasation of cancer cells into the bloodstream, their survival in circulation, infiltration into secondary sites and formation of metastatic outgrowths [192]. In the human body, cancer cells experience two fundamental types of shear stress due to the flow of bodily fluids: shear stress, generated by interstitial fluid flow within the primary TME, and shear stress, generated by the blood flow within the circulatory system [193, 194]. Here, our attention will be directed towards the impact of fluid shear stress on tumor progression both in the primary TME as well as in the bloodstream.

### Fluid shear stress exposure in tumor microenvironment

The TME can be considered as a dynamic niche where cells are constantly encountering physical and mechanical cues. Cancer cells in the TME are subjected to shear stress and hydrostatic pressure generated by interstitial fluid flow, tensile and contractile forces [195]. Interstitial fluid flow arises from the movement of fluids through the ECM, commonly occurring between blood and lymphatic vessels [196]. A common characteristic of progressing cancers is an increase in intratumoral pressure and the consequent elevated interstitial flow from the tumor mass to the adjacent healthy stroma [197]. The observed changes are the result of various contributing factors, such as vascular abnormalities, lymphatic co-option, and an increase in cell number and density. Elevated interstitial fluid and blood/lymph flows within the primary tumor produce a constant fluid shear stress, ranging between 0.1 and 1 dyn/cm^2^, that can affect cell behavior [198]. For example, several studies have shown that physical cues in the primary tumor can promote migration and invasion of cancer cells [199–201]. Shear stress can stimulate the formation of circular dorsal ruffles, F-actin rich membrane structures, thereby promoting migration of breast cancer cells [202]. In an in vitro experimental setup, oral squamous cell carcinoma cells were subjected to conditions mimicking various levels of interstitial fluid pressure, by using a humidified pressure chamber with cyclic pressure system connected to an air pump to tightly control atmospheric pressure on cells. This revealed that, as interstitial fluid pressure elevates, cell proliferation, survival and invasion capabilities of cancer cells increase [203]. A study where breast cancer and melanoma cells were seeded into a collagen-Matrigel matrix and placed within a flow chamber has demonstrated that physiological levels of interstitial flow strongly induce their migration through autocrine C-C motif chemokine receptor 7 (CCR7) signaling [204]. Multiple glioma cells lines exposed to pressure-driven flow (mimicking interstitial flow) have increased cell invasion capabilities driven by CXCR4-dependent mechanism [205].

Furthermore, increased interstitial fluid causes a decrease in transcapillary transport, leading to decreased anti-cancer drug uptake [206]. A study investigating interstitial fluid pressure in patients with metastatic melanoma has shown that interstitial fluid pressure levels significantly increases over time in patients that are not responding to immunotherapy, whereas they decreases in responders [207]. Moreover, clinical data on cervical cancer has shown that patients with higher interstitial fluid pressure are significantly more prone to develop distant metastasis and recurrence [208].

### Fluid shear stress exposure in vascular microenvironment

Not only in the primary tumor, but also during their hematogenous dissemination, cancer cells are notably exposed to circulatory fluid shear stress, the most prominent mechanical force in the bloodstream [209]. Circulatory fluid shear stress arises due to tangential frictional forces generated by blood flow acting on the surface of cells. Upon intravasation, cancer cells experience various levels of shear stress (ranging 1–30 dyn/cm^2^) [210], depending on the vessel type. Higher levels of shear stress are imposed in arteries (15–30 dyn/cm^2^) and in capillaries (10–20 dyn/cm^2^), whereas in aorta, vena cava and veins shear stress levels are lower (< 10 dyn/cm^2^) [211]. However, in the vicinity of vessel bifurcations at the heart, shear stress levels can raise up to ~ 3350 dyn/cm^2^ [212]. Fluid shear stress can be studied with various technologies both experimentally and computationally, including parallel plate flow chambers, microfluidic devices, syringe/peristaltic pumps and computational fluid dynamics modelling [213–216]. For instance, a recent study investigated the effect of brief pulses of high-level shear stress on prostate cancer cells by using a syringe pump to mimic high shear stress values in the turbulent flow region around the heart. They demonstrated that cancer cells activate RhoA-myosin II and stiffen in response to shear stress. Moreover, shear stress triggers the elevation of cortical F-actin, which further contributes to cell stiffness [214]. In another report, using a peristaltic pump to imitate arterial levels of shear stress, including high levels achieved during exercise, it has been shown that high shear stress (60 dyn/cm^2^) destroys over 90% of CTCs derived from multiple types of cancer within 4 h of circulation [213]. In a different study, computational fluid dynamics simulations were performed to mathematically predict the behavior of CTC clusters under various levels of shear stress [215]. Tumor cell intravasation is more likely to occur in areas with reduced fluid flow, as low shear stress is less detrimental for tumor cells during intravasation [217]. Recently, it has been reported that human cancer cells avoid high shear stress regions in the process of intravasation through the activation of transient receptor potential cation channel, subfamily M, member 7, also known as TRPM7 channel, a key shear-stress sensor, which promotes calcium influx into the cell followed by RhoA/myosin-II and calmodulin/IQGAP1/ Cdc42 pathway activation, subsequently reversing migration [217]. Even though carcinoma cells have higher mechanical resistance compared to non-transformed epithelial cells, shear stress in the blood circulation can readily destroy many of the intravasated cancer cells, explaining the relative inefficiency of the metastatic process [198]. Arterial levels of shear stress (15–30 dyn/cm^2^) could also increase ROS, which impair mitochondria activity and lead to further cellular death [218]. While shear forces are thought to drastically reduce the viability and number of CTCs in the circulation, a small fraction of these are mechanically robust and develop mechano-adaptive resistance. Fluid shear stress also involves human Piezo type mechanosensitive ion channel component 1 (PIEZO1) activation, which leads to the influx of extracellular calcium [219, 220]. Among other signals, PIEZO1 activates Akt/mTOR (mammalian target of rapamycin) pathway in breast cancer, and may subsequently positively regulate cell motility and survival [221, 222]. Although regulation of cytoskeletal organization and certain mechanosensitive ion channels such as PIEZO1 and TRPM7 have been shown to play a role in shear stress related responses, further mechanisms are likely to be involved.

Along with the mechanical trapping, blood flow is also a regulator of intravascular arrest of CTCs. It has been shown that early arrest on the vascular endothelium is mediated by integrin αvβ3 and CD44. Moreover, during the stronger adhesion to endothelium, cancer cells utilize integrin α5β1-dependent adhesions, which facilitate the adherence of CTCs to low shear stress regions of the vascular wall [223]. Due to the vessel diameter and numerous bifurcations, capillary beds seem to be one of the most common locations of CTC entrapment. When CTCs come across a capillary, they either become trapped and exit the vessel (in a process called extravasation) or they pass through the capillary without getting trapped. While CTCs are transiting or arrested in capillaries, shear forces can induce morphological changes and cause severe cell deformation and, hence, affect cell fate [224]. During CTC squeezing through tight capillary constrictions, cytoplasmic deformation activates several mechanotransduction signaling pathways, including yes-associated protein 1 / transcriptional co-activator with PDZ-binding motif (YAP/TAZ) and GTPase RhoA. Upon activation of Rho-family GTPase RhoA, several downstream effectors are triggered, including ROCK. As a result, ROCK promotes the activation of myosin II molecular motor, responsible for cytoskeletal rearrangement *via* contraction of actomyosin networks and can further enhance cancer cell survival and metastatic ability [225, 226].

### Resistance to shear stress

To resist detrimental effects of shear stress, cancer cells developed various resistance mechanisms, including travelling with companions. Although both single CTCs and CTC clusters are accountable for metastasis formation, they display different survival abilities in the circulation due to their physical characteristics [11]. A study performed using a microfluidic system to mimic capillary constrictions has revealed that CTC clusters may migrate through the capillary bed by rapidly unfolding into single-file chain-like geometry. This structural strategy facilitates their migration and substantially reduces the hydrodynamic resistance [227], however, fabricated glass capillaries may not fully recapitulate the properties that are ascribable to endothelial walls. Moreover, a recent study has revealed that shear stress disassociates the CTC clusters due to strong forces and disaggregates most of the CTC clusters in breast cancer [215]. Along the same line, it has been shown that majority of the CTC events that have been captured from the peripheral blood of patients and experimental models are single CTCs, with clusters being considered as a rarer event [11, 13]. While rare in numbers, CTC clusters have higher metastatic potential compared to single cells [11]. Compared to single CTCs, CTC clusters have upregulated expression of cell junction components, such as plakoglobin. Knockdown of plakoglobin halts the formation of CTC clusters and suppresses lung metastasis. Therefore, plakoglobin dependent intercellular adhesion within CTC clusters contributes to the multicellular nature of clusters as well as to their higher metastasis efficiency [11]. Increasing evidence points toward a clear CTC cluster survival advantage within the bloodstream, possibly due to their enhanced resistance to shear stress [224], among other factors. Various non-neoplastic elements have been suggested to facilitate CTC survival in circulation, likely impacting on shear stress resistance, among other aspects. For instance, through cytokines crosstalk within individual CTC-neutrophil clusters, neutrophils promote proliferation of CTCs while in circulation, thereby accelerating metastasis seeding [13]. Accordingly, breast cancer patients with detectable CTC-neutrophil clusters are characterized by a poor prognosis [13]. In another study employing an in vitro model of shear induced damage, investigators have shown that in the absence of platelets, ovarian cancer cells display a higher shear-induced membrane damage, suggesting that platelet adhesion may be protective in this context [228]. CAFs can also accompany CTCs on their metastatic journey in the blood, as observed in patients and experimental models [12]. An in vitro study addressed the role of CAFs in cancer cell survival under extremely high shear stress (5920 dyn/cm^2^) using a 3-D cell co-culture system. When co-cultured with prostate cancer cells, CAFs provide resistance to high shear, support tumor cell survival and promote proliferation *via *intercellular contact and soluble derived factors such as CCL2, CCL7 and CXCL5 [229]. In contrast to healthy individuals, circulating cancer-associated macrophages-like cells were isolated from the peripheral blood of breast, pancreatic and prostate cancer patients. A subset of these disseminated macrophages, so called circulating giant macrophages, is able to bind CTCs [230]. Recently, it has been discovered that bacteria also contribute to cancer cell survival in the bloodstream. Intracellular microbiota enhances the resistance of breast cancer cells to fluid shear stress by cytoskeletal rearrangements in CTCs, promoting cell viability. Particularly, bacteria are able to trigger the fluid shear stress-related pathways and regulate stress response within the circulation. Mechanistically, cytoplasmic bacteria inhibit the activation of RhoA and ROCK and, therefore, contractile forces triggered by mechanical forces can be alleviated [231]. Interestingly, circadian clock-regulated hemodynamics could also affect the survival of CTCs in bloodstream [232]. Given that the metastatic spread of breast cancer in mouse models and patients has been reported to occur mostly during the rest phase [233], other circadian clock-regulated parameters such as blood pressure, heartbeat and cardiac output could be contributing to these processes [234].

Overall, CTCs utilize various survival strategies, such as interactions with immune and stromal cells, allowing them to withstand shear stress. So far, most of the studies focused on molecular pathways related to mechanosensitive ion channels and cytoskeletal rearrangements, which have been widely acknowledged for their role in mechanotransduction. However, further unbiased mechanistic studies, such as genomic and transcriptomic analysis, may provide a broader overview of fluid-shear regulated genes and their impact on the metastatic cascade, possibly highlighting novel opportunities for intervention.

## Conclusions

We have provided an overview of the literature that impacts the ability of cancer cells to intravasate and travel in the bloodstream, particularly concerning the role of hypoxia and shear stress in these early stages of metastasis. Although the discussed large volume of experimental evidence highlights the relevance of these processes for the metastatic abilities of CTCs, several open questions remain. Considering that hypoxia is one of the triggers of CTC cluster shedding, it would be interesting to mechanistically investigate which hypoxia-related signals are critical in this context. Also, while the mechanical forces associated with fluid shear stress trigger several downstream biological events that affect the fate of cancer cells, the biological effects and consequences of shear stress exposure remain to be fully understood, particularly in physiological systems. Along these lines, the development of sophisticated microfluidics systems combined with computational simulations and in vivo experiments may provide new insights in understanding how flow dynamics and shear stress alter cell behavior and regulate metastasis. Deep insights into the mechanistic drivers of CTC intravasation and survival in circulation may highlight new opportunities for therapeutic interventions aimed at metastasis suppression.

## Data Availability

Not applicable.
